# Filaggrin and cytokines in respiratory samples of preterm infants at risk for respiratory viral infection

**DOI:** 10.1038/s41598-022-25897-6

**Published:** 2022-12-08

**Authors:** José M. Rodrigo-Muñoz, Beatriz Sastre, Laura Sánchez-García, María Luz García-García, Ersilia Gonzalez-Carrasco, Celia Fabra, Marta Gil-Martínez, Clara Lorente-Sorolla, Raquel García-Latorre, Sonia Alcolea, Inmaculada Casas, Cristina Calvo, Victoria del Pozo

**Affiliations:** 1 Department of Immunology, IIS-Fundación Jiménez Díaz, Av. Reyes Católicos 2, 28040 Madrid, Spain; 2 CIBER de Enfermedades Respiratorias (CIBERES), Madrid, Spain; 3 Neonatology Department, Hospital Universitario La Paz, Madrid, Spain; 4 Neonatology Department, Hospital Severo Ochoa, Leganés, Madrid, Spain; 5 Pediatric Infectious Diseases Department, Hospital Universitario La Paz, Fundación IdiPaz, Madrid, Spain; 6 Pediatrics Department, Hospital Severo Ochoa, Leganés, Madrid, Spain; 7 Translational Research Network in Pediatric Infectious Diseases (RITIP), Madrid, Spain; 8 CIBER Enfermedades Infecciosas (CIBERINFEC), Madrid, Spain; 9 Respiratory Virus and Influenza Unit, National Microbiology Center (ISCIII), Madrid, Spain

**Keywords:** Biomarkers, Paediatric research

## Abstract

Respiratory viral infections (RVIs) are frequent in preterm infants possibly inducing long-term impact on respiratory morbidity. Immune response and respiratory barriers are key defense elements against viral insults in premature infants admitted to Neonatal Intensive Care Units (NICUs). Our main goals were to describe the local immune response in respiratory secretions of preterm infants with RVIs during NICU admission and to evaluate the expression and synthesis of lung barrier regulators, both in respiratory samples and in vitro models. Samples from preterm infants that went on to develop RVIs had lower filaggrin gene and protein levels at a cellular level were compared to never-infected neonates (controls). Filaggrin, MIP-1α/CCL3 and MCP-1 levels were higher in pre-infection supernatants compared to controls. Filaggrin, HIF-1α, VEGF, RANTES/CCL5, IL-17A, IL-1β, MIP-1α and MIP-1β/CCL5 levels were higher during and after infection. ROC curve and logistic regression analysis shows that these molecules could be used as infection risk biomarkers. Small airway epithelial cells stimulated by poly:IC presented reduced filaggrin gene expression and increased levels in supernatant. We conclude that filaggrin gene and protein dysregulation is a risk factor of RVI in newborns admitted at the NICU.

## Introduction

Preterm and very low birth weight (VLBW) infants are highly vulnerable to respiratory viral infections (RVIs)^1^. Various surveillance studies have shown that RVIs, including those caused by respiratory syncytial virus (RSV), human rhinovirus (hRV), adenovirus (hAdV), metapneumovirus (hMPV), bocavirus (hBoV), or human coronavirus (hCoV), are very frequent in Neonatal Intensive Care Units (NICUs)^2–4^. Despite causing subtle or even asymptomatic infections, RVIs may have an important mid and long-term impact on children’s health^4,5^.

In preterm infants, immune response to RVIs and their pathophysiology are poorly understood^6^. Severe RVIs occurring in infancy have been found to be an independent risk factor for the subsequent development of asthma and recurrent wheezing^7,8^. In addition, RVIs of the immature lung may determine poorer respiratory outcomes in preterm infants^5^. During hRV infections, extremely preterm infants develop an increased airway secretion of T helper cell (Th)2 and Th17 cytokines, which is associated with higher respiratory morbidity during the first 2 years of life^6^. However, further research is needed to fully characterize preterm infants’ immune response to acute respiratory viral infections.

The role of bronchopulmonary dysplasia (BPD) as a risk factor for RVIs is also a matter of debate. Our research group has recently reported a higher rate of RVIs among preterm infants with BPD admitted to NICU^4^. Various pro-inflammatory cytokines and interleukins (ILs) have been involved in BPD pathophysiology, and as predictive markers, and thus the examination of free cytokines and molecules involved in the pathophysiology of the disease can help providing both knowledge and applicable biomarkers that may be associated with high RVI risk at NICU. Among cytokines, antiviral molecules are part of the main defense against RVIs, and include macrophage derived MIP-1α/CCL3 and MIP-1β/CCL4, or MCP1 being all of them needed for the induction of inflammatory antiviral responses in vivo by their chemoattractant effect^9,10^. Other chemokines although being inducers of immune antiviral mechanisms such as IL-17A produced by Th17 lymphocytes, or IL-1β related to NLRP3 inflammasome activation, are also part of the pathophysiology of the virus-induced disease, and thus their role as therapeutic targets should be evaluated^11,12^. Moreover, taking into account the relationship between RVIs and long-term respiratory morbidity, RANTES/CCL5, also known as CCL5, a molecule both related to lymphocyte and eosinophil recruitment is also a cytokine of interest in this pathology due to its role as chemoattractant in virus infections^13^. As comes to the regulation of oxygen homeostasis, HIF-1α is the master regulation of this process and it has been described that reduction of HIF-1α enhances the influenza A virus replication, and thus, is another factor involved BPD disease^14^.

Besides the inflammatory response, the epithelial barrier is the other principal component of antiviral defense. The lung barrier prevents the access of the inspired molecules and contents into the subepithelium, and is comprised of a mucociliary system of epithelial and secreting cells united by intercellular junctions including tight junctions, adherens junctions, and desmosomes^15^. The utilization of biomolecules released after epithelial barrier disintegration is an indirect methodology that allows evaluating lung integrity without the difficulty and ethical considerations of obtaining lung tissue samples from newborns. In this sense, filaggrin, a protein that belongs to the keratin protein family whose reduction has been previously associated to airway hyperresponsiveness due to skin barrier dysfunction^16^. Moreover, filaggrin deficiency has been associated to the increase of epithelial permeability and with higher IL-33/TSLP expression as a Th2 positive feedback inflammatory loop^17^, and thus, the measurement of free filaggrin in nasopharyngeal aspirates may represent an indirect measure of lung integrity. Other markers such as vascular endothelial growth factor (VEGF) and soluble VEGF receptor 1 (VEGFR1)^18,19^ have been involved in BPD pathophysiology and were described as predictive markers of abnormal development of the lung blood-air barrier, possibly promoting viral infections of the lung in NICUs^20^. The role of desmogleins as part of desmosomes are also of notorious importance in determining the integrity of the lung epithelium, to achieve insight into cell to cell cohesion, while also, calpains can give information on cytoskeletal arrangements and signal transduction of cells in the lung^21,22^. Altogether, with the study of the viral specific endosomal receptors present in the cells of the airways such as TLRs 3 and 7^23^, there is a big chance to comprehend why some patients develop RVIs in the NICU and why others do not.

We hypothesize that basal immune response and gene expression differ between infected and non-infected infants, and therefore these molecular differences might be useful as risk factors biomarkers to identify infants with more likelihood to develop an RVI at the NICU.

The aim of this study is an in-depth investigation of two components of RVI pathophysiology: first, we will characterize local immune response in respiratory secretions of preterm infants diagnosed with RVI during NICU admission; secondly, we will examine the expression and synthesis of lung barrier regulators, both in respiratory samples and in vitro models. This approach could help to understand the mechanisms under the immune modulations and the discovery of risk factors of RVIs at the neonatal ICU.

## Results

### Clinical characteristics of the study cohort

**Figure 1:**
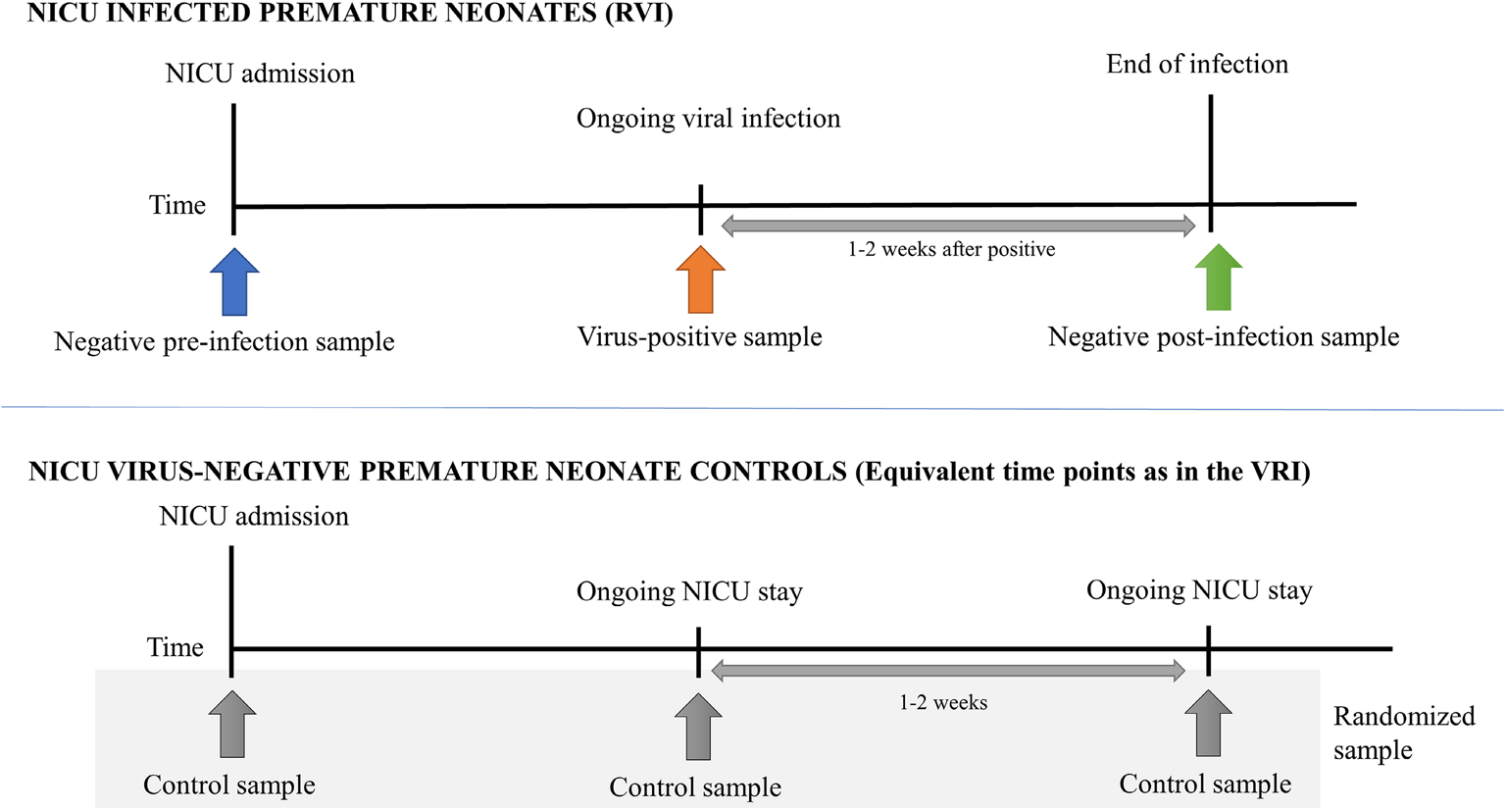
Graphical representation of the study design, representing how sampling was performed for both the NICU infected premature infants and the NICU virus-negative premature infants (control group).

Forty-nine preterm infants below 32 weeks’ gestation were included in our study. The study design is depicted in Fig. 1.

Relevant clinical data are displayed in Table 1, showing no differences between the RVI (n = 26 infants) and the negative control (n = 23 infants) group regarding the most significant neonatal clinical outcomes (*P* > 0.05 for all comparisons). Nine out of the 26 infants (35%) who suffered RVI associated clinical signs of respiratory stagnation or deterioration, as defined in the methods section.

**Table 1:** Demographic and relevant clinical data of the study population (RVI group and controls).

	Positive cases (RVI) N = 26	Negative controls N = 23	P	hRV N = 7	hAdV N = 6	hCoV N = 5	RSV N = 4	hBoV N = 3
GA (week), median (IQR)	29.6 (27.9–30.9)	29.1 (26.4–30.1)	0.138	30 (27.6–30.6)	31.1 (27.3–39.9)	28.0 (26.8–28.7)	30.9 (29.7–30.9)	31 (28.6–31)
BW(g) median (IQR)	1249 (1127–1503)	1100 (940–1460)	0.224	1290 (1065–1584)	1495 (1196–1575)	944 (917–1197)	1275 (1250–1412)	1150 (930–1150)
Male, n (%)	18 (69%)	16 (70%)	0.980	5 (71%)	3 (50%)	3 (60%)	4 (100%)	2 (67%)
BPD, n (%)	10 (42%)	10 (44%)	0.900	1 (14%)	3 (50%)	4 (80%)	–	2 (67%)
Supplementary oxygen (days), median (IQR)	19.5 (1–45.2)	20 (3–48)	0.721	3 (1–35)	31.5 (1.7–74.7)	39.0 (27.7–72.5)	2.5 (1.0–22.7)	1
MV (invasive or non-invasive) (days), median (IQR)	7 (1–21)	6 (3–16)	0.548	3 (1–27)	6.5 (1.7–20)	19.0 (7.5–40.5)	2.5 (1.0–22.0)	1
Oxygen at discharge n (%)	2 (7.7%)	2 (8.7%)	0.906	1 (14%)	1 (17%)	–	–	–
Days to reach full enteral nutrition median (IQR)	7 (5.7–10.5)	9 (7–15)	0.382	6 (5–12)	7.5 (5.7–23.2)	9 (6.5–11)	7 (6.2–22.0)	5 (4–5)
Length of stay (days), median (IQR)	59 (37–71)	61 (48–81)	0.327	38 (36–60)	62.5 (32.5–96.5)	67 (62.5–83.0)	49 (39–68)	41 (36–41)

*GA* gestational age, *wk* weeks, *BW* birth weight, *BPD* bronchopulmonary dysplasia, *g* grams, *IQR* interquartile range, *MV* mechanical ventilation.

The type and frequency of respiratory viruses detected were: hRV (n = 7; 27%), hCoV (n = 5; 19%), hAdV (n = 6; 23%; one in coinfection with hBoV and another with picornavirus hPIV), hBoV (n = 3; 12%), RSV (n = 4; 15%; one of them with hMPV) and one case of enterovirus. We further classified patients regarding the virus type, but due to the small sample size of each virus and data from each group, we cannot observe any statistical difference for any of the clinical parameters (Table 1).

### Cytokine profile of NPA supernatants is different in infants who will not develop a RVI at NICU compared who those who are infected

**Figure 2:**
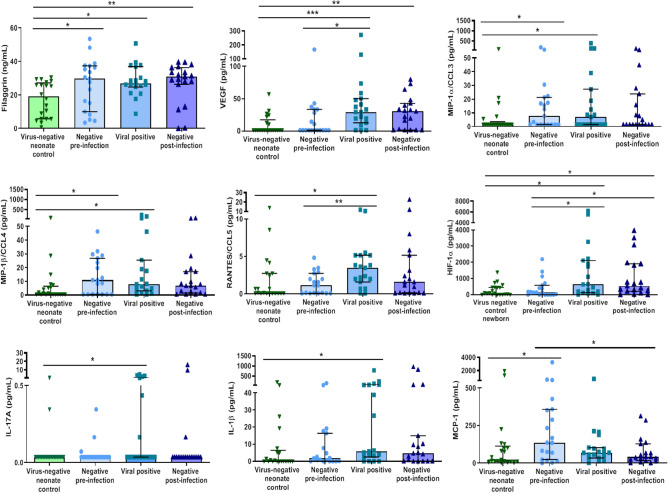
Virus-negative neonate controls have a different molecular profile in their airways compared to the pre-infected and infected neonates. The figure shows protein quantities of filaggrin, VEGF, MIP-1α/CCL3, MIP-1β/CCL4, RANTES/CCL5, HIF-1α, IL-17A, IL-1β, and MCP-1 in samples from virus-negative controls (*n* = 21) or negative pre-infection (*n* = 19), viral positive (*n* = 19), and negative post-infection samples from virus-positive premature neonates (*n* = 19) measured by ELISA or Luminex technology. Inter-group analysis was performed using Kruskal–Wallis test with multiple comparisons followed by Dunn’s uncorrected post-test. Bar graphs represent median and interquartile ranges. *P < 0.05; **P < 0.01 and ***P < 0.001.

When comparing the samples of virus-negative controls against negative pre-infection infants, we observed that several molecules were reduced (Fig. 2), including filaggrin (19.3 ± 21.5 vs*.* 29.8 ± 27.3 ng/mL; *P* = 0.02), MIP-1α/CCL3 (1.6 ± 2.0 vs*.* 7.7 ± 19.7 pg/mL; *P* = 0.03), MIP-1β/CCL4 (1.2 ± 6.0 vs*.* 10.9 ± 26.3 pg/mL; *P* = 0.04) and MCP-1 (23.1 ± 102.7 vs*.* 136.7 ± 335.0 pg/mL; *P* = 0.02). When we classified subjects of study accordingly to sex, there is no clear pattern of cytokine differentiation, showing no sex-dependent differences (Supplementary Fig. S1). Moreover, we classified samples from viral positive subjects at the time of infection, and we could not observe any difference related to virus difference for any of the molecules analyzed (Supplementary Fig. S2), being sample size and data from each group a limitation.

We went on to analyze whether the differentially expressed cytokines could be used as markers for predisposition to viral infection, or as markers for viral infection itself. As can be observed in Table 2, the levels of filaggrin, MIP-1α/CCL3 and MCP-1 could be used as moderate predisposition biomarkers with AUC values > 0.68, with the following values of specificity (> 84%) and sensitivity (> 53%). In addition, the levels of filaggrin, VEGF, MIP-1β, RANTES/CCL5, HIF-1α and IL-1β were identified as infection biomarker molecules with AUC values > 0.72, having the next values of specificity (> 79%) and sensitivity (> 55%) (Table 2).

**Table 2:** Diagnostic value of supernatant molecules to differentiate between virus-negative premature controls against pre-infection premature infants and against those with ongoing infection.

Individual molecule	AUC (CI)	P value	Cutoff (units)	Sensitivity (%)	Specificity (%)
**Viral infection predisposition biomarker**					
Filaggrin (ng/mL)	**0.69 (CI = 0.52–0.87)**	** 0.04**	**29.7**	**53**	**95**
HIF-1α (pg/mL)	0.54 (CI = 0.35–0.71)	0.69	122	63	60
VEGF (pg/mL)	0.56 (CI = 0.39–0.75)	0.44	27.7	32	90
RANTES/CCL5 (pg/mL)	0.56 (CI = 0.38–0.74)	0.52	0.92	42	35
MIP-1α/CCL3 (pg/mL)	**0.68 (CI = 0.51–0.85)**	** 0.05**	**7.54**	**53**	**85**
MIP-1β/CCL4 (pg/mL)	0.66 (CI = 0.48–0.84)	0.09	8.13	63	85
IL-1β (pg/mL)	0.60 (CI = 0.41–0.77)	0.25	0.12	26	45
MCP-1 (pg/mL)	**0.70 (CI = 0.52–0.86)**	** 0.02**	**134**	**58**	**84**

Logistic regression model	AUC (CI)	AIC	Cutoff (units)	Sensitivity (%)	Specificity (%)
Filaggrin + MCP-1 + IL-1β (Categorical)	0.72 (CI = 0.54–0.90)	48.2	0.43	68	85

Individual molecule	AUC (CI)	P value	Cutoff (units)	Sensitivity (%)	Specificity (%)
**Active infection biomarker**					
Filaggrin (ng/mL)	**0.76 (CI = 0.61–0.91)**	** 0.005**	**20.7**	**90**	**55**
HIF-1α (pg/mL)	**0.74 (CI = 0.57–0.89)**	** < 0.05**	**115**	**79**	**60**
VEGF (pg/mL)	**0.80 (CI = 0.66–0.94**	** 0.001**	**5**	**84**	**65**
RANTES/CCL5 (pg/mL)	**0.72 (CI = 0.56–0.89)**	** 0.01**	**0.41**	**90**	**55**
MIP-1α/CCL3 (pg/mL)	0.66 (CI = 0.49–0.83)	0.07	6.32	53	80
MIP-1β/CCL4 (pg/mL)	**0.75 (CI = 0.59–0.90)**	** 0.008**	**1.33**	**84**	**60**
IL-1β (pg/mL)	**0.72 (CI = 0.56–0.88)**	** 0.01**	**1.65**	**79**	**65**
MCP-1 (pg/mL)	0.61 (CI = 0.47–0.80)	0.13	28.5	16	45

Logistic regression model	AUC (CI)	AIC	Cutoff (units)	Sensitivity (%)	Specificity (%)
Filaggrin + RANTES + MIP-1β + MCP1 (Continous)	0.80 (CI = 0.65–0.94)	43.1	0.62	74	90

Significant values are in bold.

*AUC* Area under the curve, *AIC* Akaike information criterion, *CI* confidence interval.

Moreover, we performed logistic regression multivariable models where we combined the biomarker value of the different measured cytokines, and we observed that the combination of the categorical values of filaggrin, MCP-1 and IL-1β (applying Youden index cutoff values) is a good biomarker for viral infection predisposition, having an AUC of 0.72, with 68% of sensitivity and 85% of specificity, that can help in differentiating neonates with RVI risk at NICU (Table 2). Similarly, the logistic regression model using continuous values of filaggrin, RANTES/CCL5, MIP-1β**/**CCL4 and MCP1 has an AUC of 0.80, with 74% of sensitivity and 90% of specificity for discrimination of those neonates who are infected at the moment of analysis at the NICU (Table 2). The graphical representation of the ROC curves for both individual molecules and logistic regression models are provided in the Supplementary Fig. S3.

### The immune response of infants with RVI changes with the course and evolution of the infection

Different NPA samples (negative pre-viral infection, positive viral (ongoing infection) and negative post-infection samples) were used for immune-evolution analysis. Regarding the differences between negative pre-infection samples and positive viral samples (ongoing infection) an infection caused increase of RANTES (3.5 ± 3.6 vs. 1.2 ± 3.8 pg/mL; *P* = 0.02); VEGF (1.2 ± 32.5 vs. 29.4 ± 37.4); and HIF-1α (638.6 ± 1989.7 vs. 145.4 ± 584.0 pg/mL; *P* = 0.03) was observed, as depicted in Fig. 2, reflecting the expected increase in immune molecules caused by a viral infection. Interestingly, filaggrin levels did not vary over the course of infection in the RVI infants (*P* > 0.05), which could reflect a permanent dysregulation of this protein in RVI infants compared to controls.

Finally, HIF-1α was increased in negative post-infection samples compared to negative pre-infection samples (542 ± 1697.5 vs*.* 145.4 ± 584.0 pg/mL; P = 0.04; Fig. 2), while the opposite trend was observed for MCP-1 (41.3 ± 111.4 vs. 136.7 ± 335 pg/mL; *P* = 0.02). A non-significant decrease in MIP-1α/CCL3, MIP-1β, RANTES/CCL5 and MCP-1 (Fig. 2) was observed in negative post-infection samples, which could be related to the resolution of the inflammatory response.

### Filaggrin cellular gene expression frequency and protein levels are altered in the NPA of neonates who will in time became infected by RVIs in NICU

**Figure 3:**
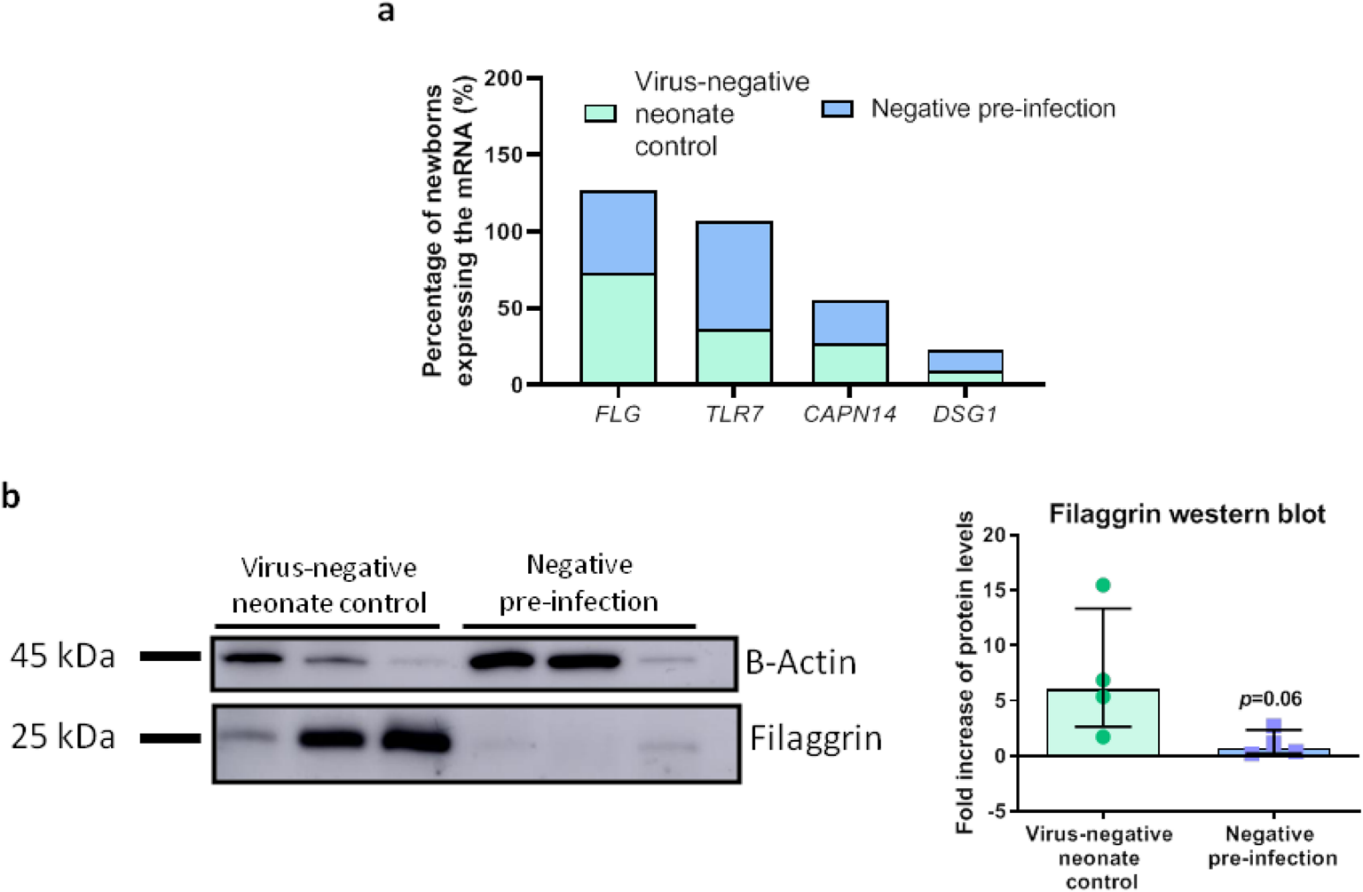
Gene expression and protein quantity of filaggrin in the NPA cells from neonates differs in virus-negative neonate controls compared to samples from negative pre infection subjects. (**a**) Percentage of neonates (negative pre-infection samples from virus-positive [*n* = 7–11] and from virus-negative controls [*n* = 11–19]) with mRNA expression in NPA cells measured by qPCR. Frequency analyses were performed using Fisher’s exact test. (**b**) Quantity of filaggrin in NPA cells from neonates measured by Western blot (*n* = 4 for each group). Inter-group analysis was performed using Mann–Whitney U test. Bar graphs represent percentage (**a**) or median and interquartile ranges (**b**). *kDA* Kilodaltons.

To obtain information about the basal state (at the moment of NICU admission) of the airway barrier, we compared negative pre-infection samples from RVI and from virus-negative control groups. Having observed in our previous results that free filaggrin levels are different in samples depending on if the neonate becomes infected or not, we next studied the frequency of neonates who basally (never infected and pre-infection) do express filaggrin gene (*FLG*) at NPA cellular level, in order to get an insight of its expression in the cellular compartment of the airways. Moreover, we analyzed filaggrin protein quantity in the cells obtained from the NPAs using western Blot. We also studied the frequency of infants expressing other relevant genes such as *TLR7, CAPN14 and DSG1* to get more insight into the state of the barrier. As depicted in Fig. 3a, the frequency of neonates with airway expression of *FLG* was reduced in the pre-infection samples from the RVI positive (55%) compared to the negative control (74%) group; the opposite occurred for *TLR7,* where higher rates of expression were found in the RVI positive pre-infection (71%) than in the negative control (37%) group. Neither of these trends reached statistical significance (*P* > 0.05), although these differences may provide information about how is the expression of immune and barrier genes in the free cells of the neonate’s airways. The frequency of expression of *CAPN14* and *DSG1* was similar between the negative pre-infection and the negative control group. The lower *FLG* expression in NPA cells was associated with a reduction in protein levels, with a 6.7-fold-lower protein content in negative pre-infection samples from the RVI positive group compared to negative control group samples (*P* = 0.06; Fig. 3b). Original blots are presented in Supplementary Fig. S4.

### Epithelial cells from healthy donors increase immune-gene expression and release higher filaggrin after viral stimuli plus wounds

**Figure 4:**
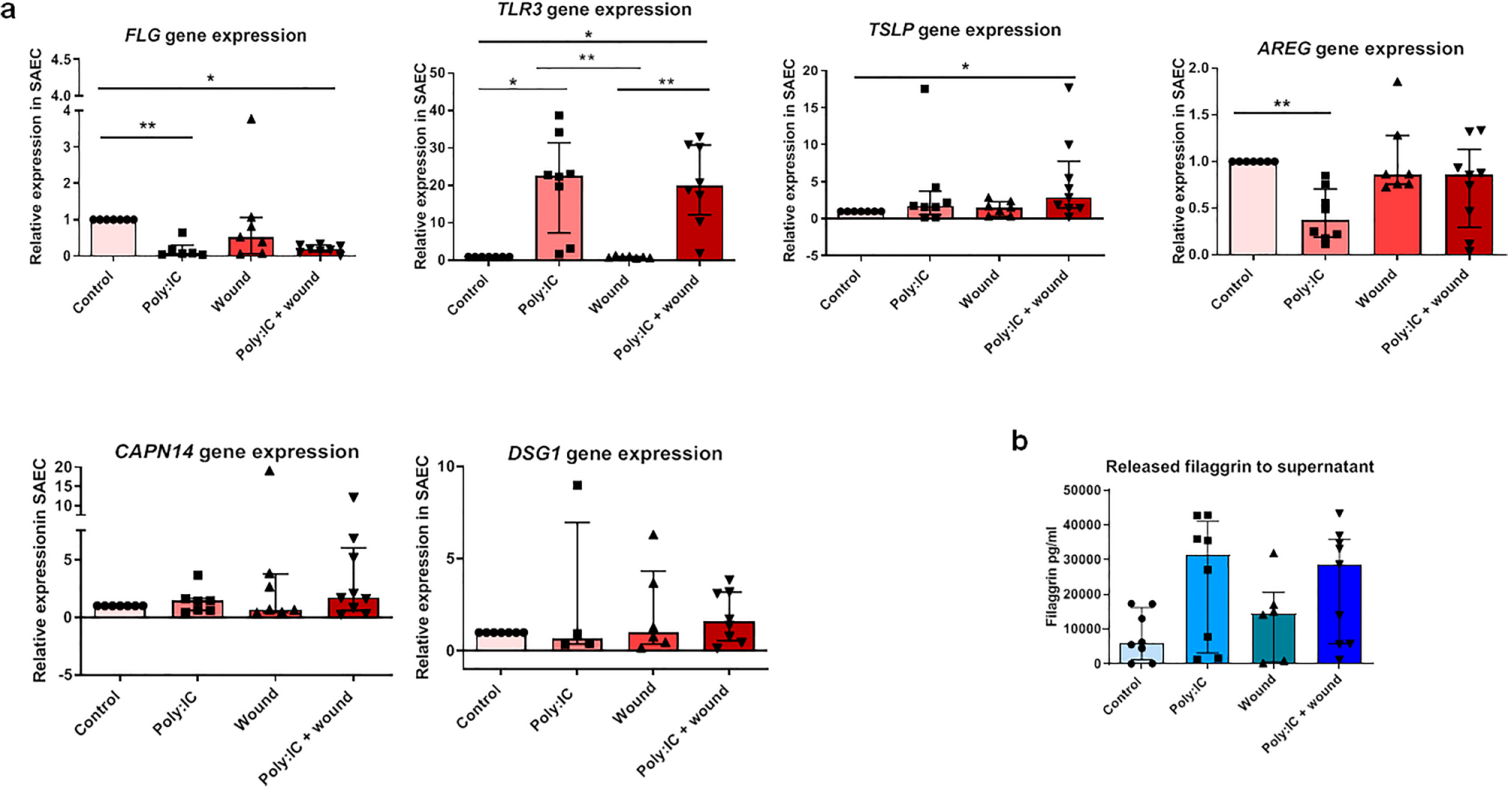
Filaggrin is altered in SAEC by viral stimuli and wounds in the monolayer. (**a**) Gene expression of *FLG* (*n* = 6–8), *TLR3* (*n* = 6–8), *TSLP* (*n* = 7–9), *AREG* (*n* = 7–9), *CAPN14* and *DSG1* (*n* = 6–9) (relative expression as 2^−∆∆Ct^) measured by qPCR and (**b**) filaggrin (pg/mL) protein synthesis measured by ELISA in SAEC treated with 20 ng/mL of poly:IC (polyinosinic:polycytidylic acid), wounded, or treated with poly:IC and wounded for 24 h (*n* = 5–9). Inter-group analysis was performed using the Kruskal–Wallis test with multiple comparisons test followed by Dunn’s post-test. Bar graphs represent median and interquartile ranges. *SAEC* small airway epithelial cells. *P < 0.05; **P < 0.01.

To study lung barrier status we used a RVI in vitro model of SAEC, using poly:IC (20 ng/mL) as viral stimuli^24^. We observed a reduction in *FLG* gene expression in SAEC with (relative expression = 0.19 ± 0.19) or without (relative expression = 0.07 ± 0.26) wounds compared to the untreated control; (*P* = 0.004 and *P* = 0.02 respectively; Fig. 4a). Regarding the cell culture supernatant, the opposite effect was observed, with increased, although non-significantly, levels in secreted filaggrin both in poly:IC treated (31,333 ± 37,915 ng/mL; P > 0.05) and in poly:IC plus wound (28,772 ± 30,216 ng/mL; P > 0.05) compared to controls (5950 ± 15,047 ng/mL) and with wounds (14,528 ± 20,039.4 ng/mL) (Fig. 4B).

Other molecules related to RVI and the lung barrier were studied, showing that poly:IC overexpressed *TLR3* in SAEC independently of wounds (relative expression = 22.58 ± 24.09 in unwounded and 19.85 ± 18.65 in wounded; *P* = 0.01 and *P* = 0.02 respectively), and increased *TSLP* in wounded SAECs relative expression = 2.91 ± 6.32; *P* = 0.04), while reducing *AREG* in unwounded (relative expression = 0.37 ± 0.52; *P* = 0.003), and without causing significant changes in *CAPN14* and *DSG1* (Fig. 4a).

## Discussion

This study, carried out in a population of preterm infants admitted to NICU, shows a distinct pattern of immune profile and epithelial barrier gene expression in baseline NPA from neonates who go on to develop RVI. Our findings indicate that vulnerability to infection may be related to immunological response or epithelial barrier integrity. We would like to highlight that the control group were randomized samples, obtained over the same time as the RVI positive newborns in order to obtain more representative data from this group (Fig. 1). In spite of this, we believe that modulation of the control group’s molecular and immune response to be unlikely due to the absence of RVIs. On analysis of respiratory samples from never infected infants (controls) compared to pre-infected neonates (preterm infants who went on to develop RVI) we observed several differences in immune response cytokines and other molecules (including filaggrin, MIP-1 [α/β] and MCP-1), as well as in the expression and location of filaggrin in NPAs, that may indicate a barrier-immune deconfiguration predisposing certain individuals to future infection. Differences between samples from controls and neonates with ongoing viral infection in several molecules (filaggrin, VEGF, RANTES (CCL5), HIF-1α, IL-17A and IL-1 β) were also observed, indicating an immune response associated with viral infection.

By means of in vitro experiments we replicated reduction of *FLG* expression in airway epithelial cells after exposure to viral particles, observing that the filaggrin released into the extracellular space may be related to a failure in a correct epithelization, leading to a vicious circle of barrier disruption and infection.

The lung’s immune response begins at the respiratory epithelium. Although our results are not statistically significant, we observed a modification of the frequency of gene expression and quantity of filaggrin levels in cells from the NPA of premature neonates who went on to suffer respiratory viral infection. Filaggrin is a protein that maintains the epithelial barrier of the nose and skin, and filaggrin defects have previously been associated with spontaneous atopic dermatitis, acting as disease biomarkers, and with secondary lung inflammation in mice^25,26^. Filaggrin loss of function mutations have also been associated with airway diseases, with higher allergen sensitization in children^27^ and early wheezing^28^.

Interestingly, we found differences in the expression of several immune-barrier molecules (filaggrin, MIP-1α/CCL3, MIP-1β/CCL4 and MCP-1) in NPA supernatants of virus-negative controls compared to negative pre-infection samples of RVI neonates. These differences point to a possible predisposition to infection in RVI infants characterized by modified levels of filaggrin, MIP-1α/CCL3, MIP-1β/CCL4 and MCP-1. Filaggrin, MIP-1α, and MCP-1 levels may be useful for predicting the risk of subsequent RVI during NICU admission, using the NPA obtained early after birth. As previously mentioned, filaggrin’s role is to maintain the epithelial barrier, and its release into the extracellular space can be associated with the barrier’s disruption, and the consequent increase in permeability to substances and microorganisms. MIP-1α/CCL3 is necessary for anti-viral immune responses, and as previously described is a vital molecule in the recruitment of the leukocytes as monocytes, T lymphocytes (CD8^+^ and CD4^+^) and B lymphocytes to the site of infection^29^ together with MIP-1β, which is also involved in mucosal humoral immunity, in the trafficking of T lymphocytes through the vascular endothelium and in the production of cytokines performed by T helper cells, like the release of IL-6 and IL-4^30^; while and MCP-1 has also been described as a key antiviral molecule, as seen in studies unraveling the importance of this molecule in the severe cases of COVID-19, where increase of this molecule was predictive of respiratory failure^31^.

Several immune compounds were identified in NPA supernatants of infants with ongoing RVIs which differed to those of virus-negative controls, including IL-1β, a cytokine that promotes antiviral immunity through the activation of the inflammasome complex with nuclear factor-κB, inducing immune cell trafficking, activation and cytokine production of TNF-α and IL-6, that are characteristic of antiviral defense^12,32^; VEGF and HIF-1α, both related to BDP development, as HIF-1α is an upstream regulator of VEGF, and together with NF-κβ are involved in apoptosis, alveolar structure and lung capillary density in neonatal development, factors that may differentiate between healthy and RVI predisposes neonates^33^; and finally, filaggrin, associated with epithelial barrier diseases, as previously described, being a marker of decrease of this molecule in the airway epithelium linked to higher permeability to viruses by barrier disruption^28^. Some of these molecules are indeed potential biomarkers for differentiating between infected and uninfected neonates in the NICU, as seen in the ROC curve analysis and in the logistic regression models where cytokines and molecules related to lung integrity are good biomarkers to differentiate subjects and to predict risk.

The onset of infection provokes a series of changes in the immune compound composition of the NPA. During and after infection, a further increase in VEGF and HIF-1α was observed, which may reflect enhanced angiogenesis, and possible are also related to the homeostasis of oxygen during lung infections^34^. Equally higher concentrations of IL-1β, IL-17A and RANTES/CCL5 were detected. IL-17A is involved in viral clearance in the airways, being secreted by T cells, which creates an inflammatory *milleu* that favors the action of neutrophils and NK cells^35^; and RANTES (CCL5), that causes viral replication control by the antiviral characteristics of eosinophils and T cells^13,36^. During viral immune responses, it seems that a general increase of antiviral and proinflammatory molecules like MIP-1α/CCL3 and MIP-1β/CCL4 occurs, a process that is coherent as a mechanism for virus control and lung homeostasis at the first stages of infection^30^. Interestingly, MCP-1 was reduced in the ongoing and post-infection RVI group, as described after immune recovery in bronchiolitis^37^, as a mechanism that might be related to the finalization of inflammation and antiviral responses^10^.

Several studies have described that umbilical cord blood samples from preterm infants showed downregulation of immune pathways, including complement cascades in infants and upregulation in maternal blood, which may imply that besides lung barrier integrity, immune responses might be reduced in preterm neonates^38,39^. Regarding the molecular sensors of innate immunity in premature infants, previous publications describe that TLR4 and TLR2 receptors are reduced in animals, impairing neutrophil recruitment, while neutrophils from neonates themselves show impaired migration, phagocytosis and cytotoxic activity^40–42^. Adaptative immune responses may influence the risk of viral infections in newborns as their CD4^+^ T cells have delayed synthesis of IFNγ and IL-2 in presence of viral infections^40,43^. Genetic studies can be very helpful in the determination of risk factors, such as mannose-binding lectin (MBL) mutations causing MBL deficiency, which are associated with higher risk of sepsis in preterm neonates^44^, or increased IFI27 expression, associated with higher RSV infection severity^45^.

These findings from the literature suggest that the risk of RVI in premature neonates is a complex combination of alterations in epithelial barrier integrity and immune system development. Our study provides more insight into this subject, describing a profile of NPA-specific biomarkers that could be of use in prevention and risk management of newborn ICU respiratory viral infections.

In the in vitro model of RVI we observed that, just as in NPAs from premature neonates, viral stimulus provokes a reduction in filaggrin expression, and an increase of released levels, perhaps indicating that viruses can cause barrier disruption by preventing cornification and airway homeostasis^46^, although this hypothesis warrants further experimentation. Besides, viral particles upregulated *TLR3* and *TSLP* expression as previously described in dendritic cells, where these molecules promote the differentiation of Th17 cells under Th2 polarizing conditions^24^, and in infants with bronchiolitis where this Th2 environment can lead to asthma and allergic diseases in the long term^47^. Interestingly, epithelial barrier integrity is affected by reduced *AREG* expression, a protein previously associated with tissue integrity, thus having relationship with the increase in permeability of the airway epithelium^48^. Nevertheless, we could not imitate the predisposition of infants to viral infection by wounds, as disruption of SAEC monolayer did not affect the expression of the selected genes, indicating that, in future, other models for barrier disruption would be more appropriate.

The limitations of this study consist, firstly, in the lack of airway epithelial cell samples from neonates before and after infection, to prove a direct link between the cellular models and the nasopharyngeal aspirate samples. We used a cellular model consisting in small airway epithelial cells, which are anatomically different from nasal epithelial cells, and therefore might not respond in the same manner to viral stimuli. We also randomized timing of control samples during NICU admission to obtain a group that is representative of all three states (pre-infection, active infection, and post-infection), which might mask temporal differences in the control group. Another limitation resides in the small sample size of our study, mainly due to the difficulty of obtaining samples from premature infants. Nevertheless, we feel that our results are of sufficient importance to inaugurate filaggrin in neonates as a field of research.

In summary, this study shows that preterm infants who will go on to suffer respiratory viral infection during NICU admission have a distinct respiratory epithelium immune profile at their NPA supernatants, consisting of higher baseline concentrations of immune and barrier-related molecules which can be used as possible biomarkers for infection risk for preterm neonates at NICU. In addition, the dysregulation of filaggrin synthesis and release from the epithelium, found in NPA and in SAEC with wounds and Poly:IC highlight the possible role of this molecule in epithelial barrier disruption, favoring RVI and triggering chronic diseases in neonate infants admitted in the NICU.

## Methods

### Study population

The patients included in this study are part of a prospective observational respiratory viral infection surveillance study of preterm infants (below 32 weeks of gestation) from the Departments of Neonatology of La Paz University Hospital (Madrid, Spain)^4^ and the Severo Ochoa University Hospital (Leganés, Madrid, Spain) between December 2018 and December 2020 and whose primary aim was to determine the viral etiology of respiratory infections during the NICU admission, the outcome of infected infants and the risk factors related.

The present substudy is part of the previously mentioned prospective study and has the aim to describe the local immune response and the expression and synthesis of lung barrier regulators in respiratory secretions of neonates who develop RVIs during the NICU; and in vitro models. Inclusion criteria were being a preterm neonate of less than 32 weeks of gestation who was included in the NICU. Patients older than 3 days at enrolment, those with major malformations, and those who died within the first week of life, were excluded. A flow chart with the recruitment and classification of the subjects of study is represented in the Supplementary Fig. S5. The study was approved by La Paz University Hospital Clinical Research Ethics Committee and Severo Ochoa University Hospital Clinical Research Ethics Committee (code number HULP-PI-2255). Informed consent was obtained from parents of the infants included in the study. All infants’ data were treated anonymously and codified.

### Sampling

Nasopharyngeal aspirates (NPA) for RVI surveillance and immunological analysis were collected within the first 3 days after birth and then weekly until discharge. Additional NPA were obtained in the event of respiratory symptoms or clinical suspicion of sepsis.

RVI samples were selected by identification of infants with viral infection confirmed by PCR. For each participant, a first negative pre-infection NPA sample (taken when the infant is admitted at the NICU), a second positive sample taken during ongoing viral infection, and a third negative post-infection sample (obtained at least 1 or 2 weeks after the last positive NPA) were selected for the study. The control group was formed by randomized samples (we chose the samples from the non infected controls using samples from different times at each control NICU stay, to homogenize as much as possible) from premature infants admitted to the NICU with a similar gestational age to their RVI counterparts who did not develop RVI during hospitalization, collected at three different times (equivalent to the three samples taken from patients in the RVI group, and therefore happening in the same seasons). The selection of control samples at equivalent time points was carried out to reduce the possible bias caused by differences in gestational age between groups. The study design is represented in Fig. 1.

Clinical parameters were recollected for assessment of the neonate’s state, including gestational age, birth weight, bronchopulmonary dysplasia, supplementary oxygen, oxygen at discharge, days to reach full enteral nutrition, length of stay; and finally clinical signs of respiratory stagnation or deterioration defined as no progress made in the withdrawal of respiratory support despite the passage of time, and without any other condition that justifies it and as increased need for respiratory support compared to the previous situation with no other cause, respectively.

One part of each NPA was used for viral detection and sent to the Respiratory Virus and Influenza Unit at the National Microbiology Centre (ISCIII, Madrid, Spain) where three RT-nested PCR assays capable of detecting a total of 16 respiratory viruses^49^ were performed. The other part of each NPA was separated into cells and supernatant by centrifugation. Samples comprising of a high amount of mucus were filtered with a 40 µm nylon filter. Pellet was resuspended in 0.7 mL of Qiazol Lysis Reagent (Qiagen, Hilden, Germany) and frozen at – 80 °C. Supernatants were adjusted to 1.5 mL with PBS 1X and frozen at – 80 °C.

### NPA cytokine and chemokine assays

Twenty-four cytokines and chemokines were determined in NPA supernatant using a commercial Luminex panel according to the manufacturer’s instructions (Magpix, Merck Millipore, Massachusetts, USA): transforming growth factor beta 1 (TGF-β1), IL-33, IL-25, epidermal growth factor (EGF), fibroblast growth factor 2 (FGF-2), granulocyte macrophage colony-stimulating factor (GM-CSF), interferon (IFN)-α2 and γ, IL-10, IL12p70, IL-13, sCD40L, IL17A, IL-9, IL-1β, IL-2, IL-4, IL-5, IL-8, IP-10, MCP-1, macrophage inflammatory protein (MIP)-1α, MIP-1β, and VEGF. Amphiregulin (R&D Systems, Abingdon, UK), filaggrin, hypoxia-inducible factor 1-α (HIF-1α) and HIF-2α were analyzed by ELISA Kit (Cloud-Cone Corp., Texas, USA).

### Small airway epithelial cell culture

The expression and synthesis of lung barrier regulators in the study and control group were investigated in NPA. An in vitro model was developed to further examine the results obtained in vivo, using primary small airway epithelial cells (SAEC) from healthy subjects (Lonza, Basel, Switzerland) cultured in a specific growth medium (Promocell, Heidelberg, Germany). Media were supplemented with 100 U/mL penicillin and 100 µg/mL streptomycin and maintained at 37 °C in an atmosphere containing 5% CO_2_. Cells were incubated with 20 ng/mL of poly:IC (Sigma Aldrich, MO, USA). Disruption of epithelial barrier integrity was performed using a pipette tip.

### Gene expression assays

RNA from the cellular fraction of NPAs and SAECs was obtained using the phenol–chloroform technique from QIAzol (Qiagen)^50^. 500 ng of RNA quantified by Nanodrop ND-1000 spectrophotometer (Thermo Fisher Scientific, Waltham, MA, USA) was reverse-transcribed with a High-Capacity cDNA Reverse Transcription Kit (Applied Biosystems, Foster City, CA, USA), and analyzed by semi-quantitative real-time PCR (qPCR) on a StepOnePlus Real-Time PCR System with TaqMan™ gene expression probes (Applied Biosystems) for *18s, TSLP, TLR7, TLR3, FLG, AREG, DSG1, CAPN14* and TaqMan™ Gene Expression MasterMix (Applied Biosystems). Relative gene expression was calculated using the Cycle Threshold (Ct) and the 2^−ΔΔCt^ method^51^, where:

$$\Delta \Delta {\text{Ct}} = \Delta {\text{Ct}}_{{{\text{population1}}}} {-}\Delta {\text{Ct}}_{{{\text{population2}}}} \,{\text{and}}\,\Delta {\text{Ct}} = \Delta {\text{Ct}}_{{{\text{gene}}}} {-}\Delta {\text{Ct}}_{{{\text{Housekeeping}}\,{\text{gene}}}} .$$

### Protein analysis by Western blot

Proteins from the cellular fraction of NPAs were extracted from QIAzol using chloroform-isopropanol and 1% SDS. Proteins were quantified by BCA (Thermo Fisher Scientific) and resolved (10 µg) in Acrylamide/Bis-acrylamide gels. They were then transferred to polyvinylidene difluoride (PVDF) Amersham HybondTM-P membranes (GE Healthcare, Buckinghamshire, UK) and blocked 2 h in 1× PBS/5% non-fat-dried milk/0.2% Tween-20 at room temperature. Overnight incubation was performed at 4 °C with antibodies against β-actin (Cell Signaling Technology Cat# 4970, RRID:AB_2223172, Leiden, The Netherlands, 1:1000) and filaggrin (Novus Biologicals, LLC, CO, USA, 1:500) in 1× PBS/0.5% non-fat-dried milk/0.2% Tween-20, and further incubation with the secondary anti-rabbit (Millipore Cat# AP156P, RRID:AB_11213985, Burlington, MA, USA, 1:1000) or anti-mouse (Thermo Fisher Scientific, 1:1000) antibody-HRP for 2 h at room temperature. Immobilon^®^ Crescendo Western HRP Substrate was used, with visualization using an Amersham Imager 600 (GE Healthcare). Data were curated using Quantity One 1-D software (Bio-Rad).

### Statistical analysis

Descriptive data were expressed as median and interquartile ranges for continuous variables, and percentages for categorical variables. Normal continuous variables were compared using ANOVA with the Bonferroni correction, or through T-tests; and the Mann–Whitney U test or the Kruskal–Wallis test with uncorrected Dunn’s post-test was used for non-normal samples. Categorical variables were compared using a chi-squared test or Fisher’s exact test. P-values < 0.05 were considered statistically significant. The Youden index was used to set the optimal cut-off point. Logistic regression models were performed to determine the possibility of using a combination of molecules in order to differentiate between conditions, using as variables either the continuous data, or the categorical classification using as cutoff values the ones determined by the Youden index. The Akaike information criterion (AIC) was used for the determination of the best fitting classification models. Analyses were carried out using Graph-Pad Prism 8 (San Diego, CA, USA), and multivariate logistic regression models were performed using R (https://www.r-project.org/).

### Ethics statement

Subject’s parents signed the required informed consent. The study was conducted in accordance with the principles of the Declaration of Helsinki and was approved by the hospital’s ethics committee.

## Supplementary Information

Supplementary Information.

## Data Availability

The data that support the findings of this study are available from the corresponding author, V.P., upon reasonable request.
