# Supplementary material for: Filaggrin and cytokines in respiratory samples of preterm infants at risk for respiratory viral infection
Source: Sci Rep. 2022 Dec 8;12:21278. doi: 10.1038/s41598-022-25897-6 (PMC9731953; doi:10.1038/s41598-022-25897-6)
Supplement: Supplementary file 1 — Supplementary Information. [file 41598_2022_25897_MOESM1_ESM.docx]

**
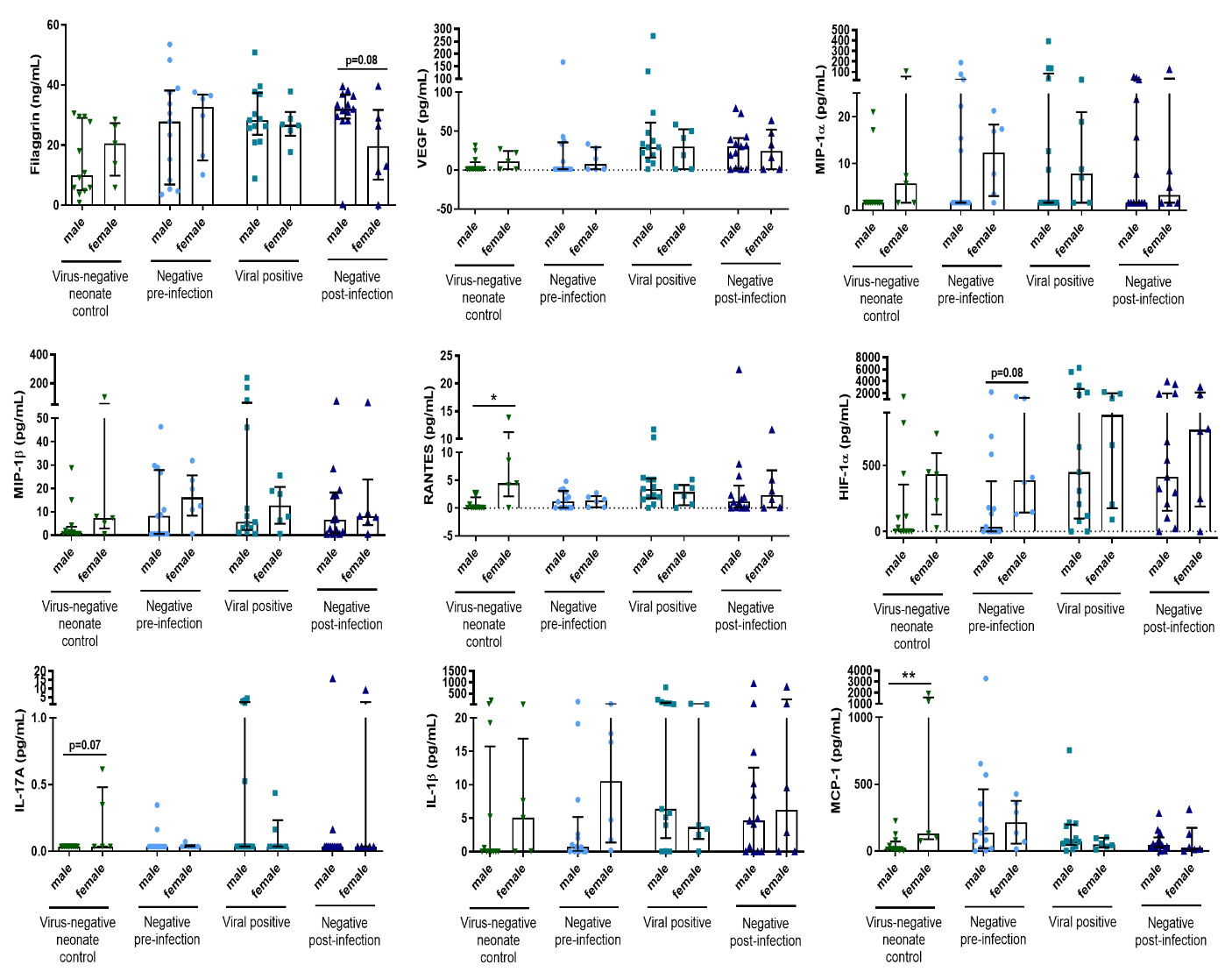
Supplementary figure S1. Sex does not determine cytokine differences in the NPA supernatants from neonates included in the NICU**.


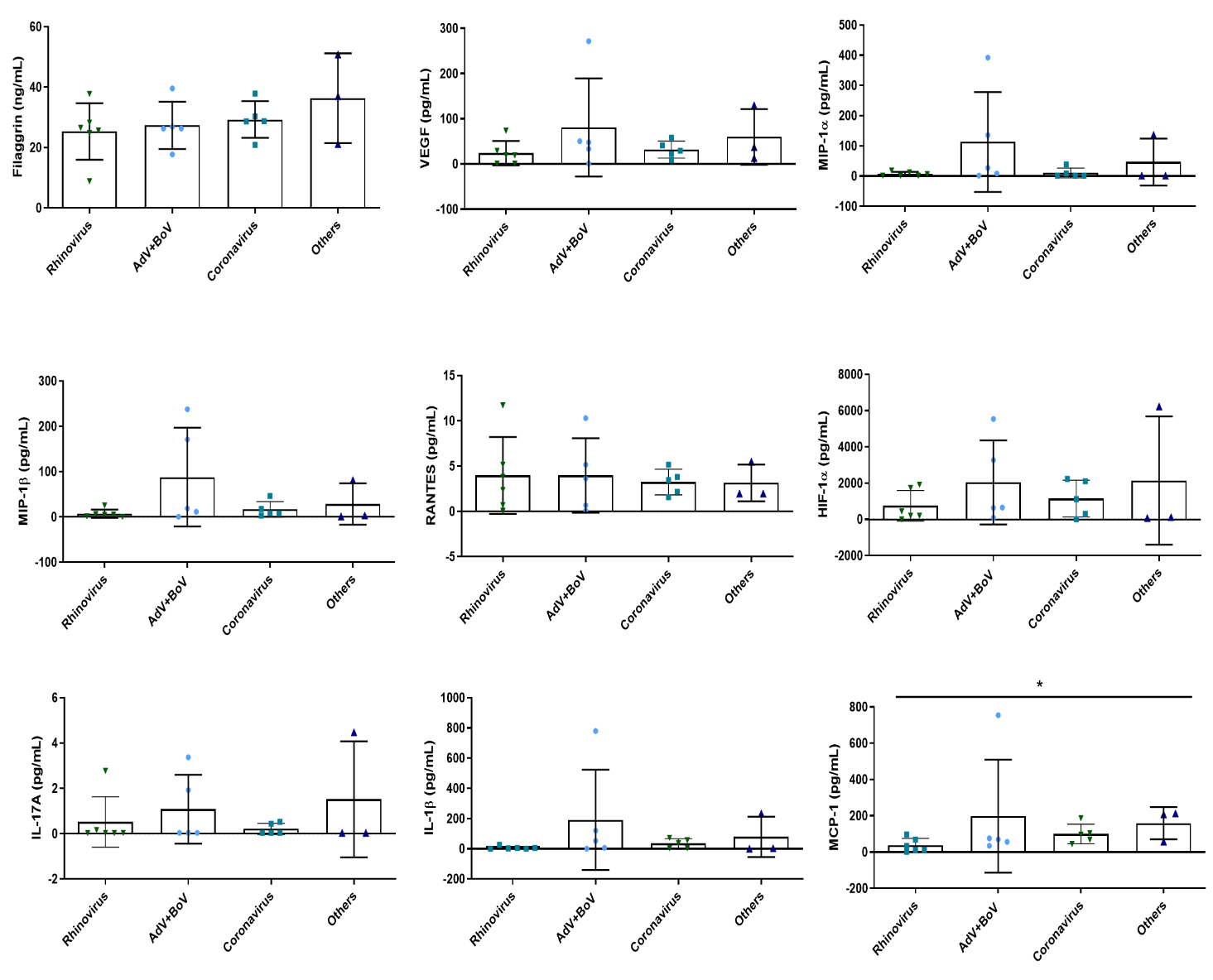
**Supplementary figure S2. Viral entity infection does not affect cytokine secretion in the NPA supernatants from neonates included in the NICU**.

**
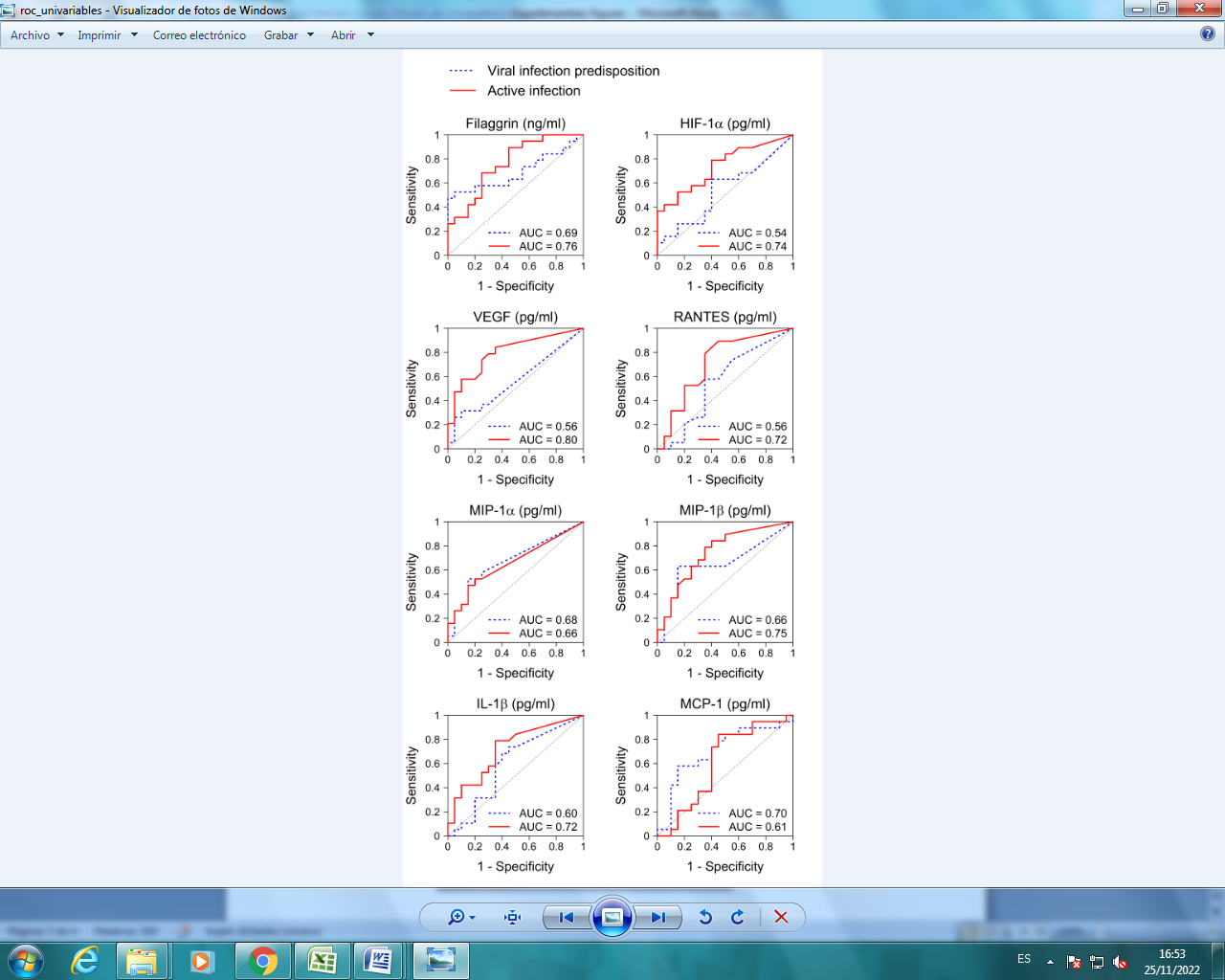
Supplementary figure S3. AUC curves graphic representation of the studied biomarkers, both individual molecules and the elaborated logistic regression models.**

A


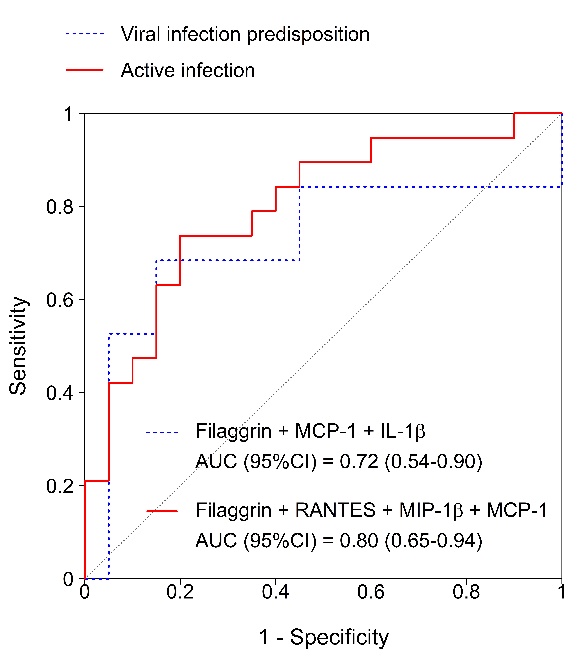


B

**Supplementary figure S4. Original western blot images.**


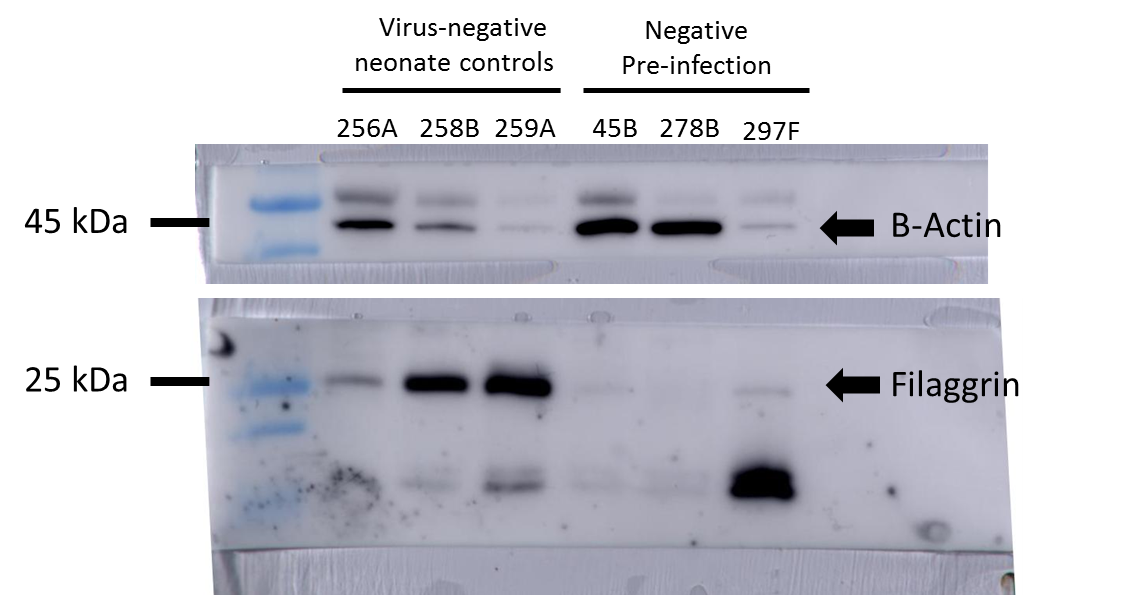


NOTE: the crop at the right is due to the limits of the Amersham Imager 600 (GE Healthcare).


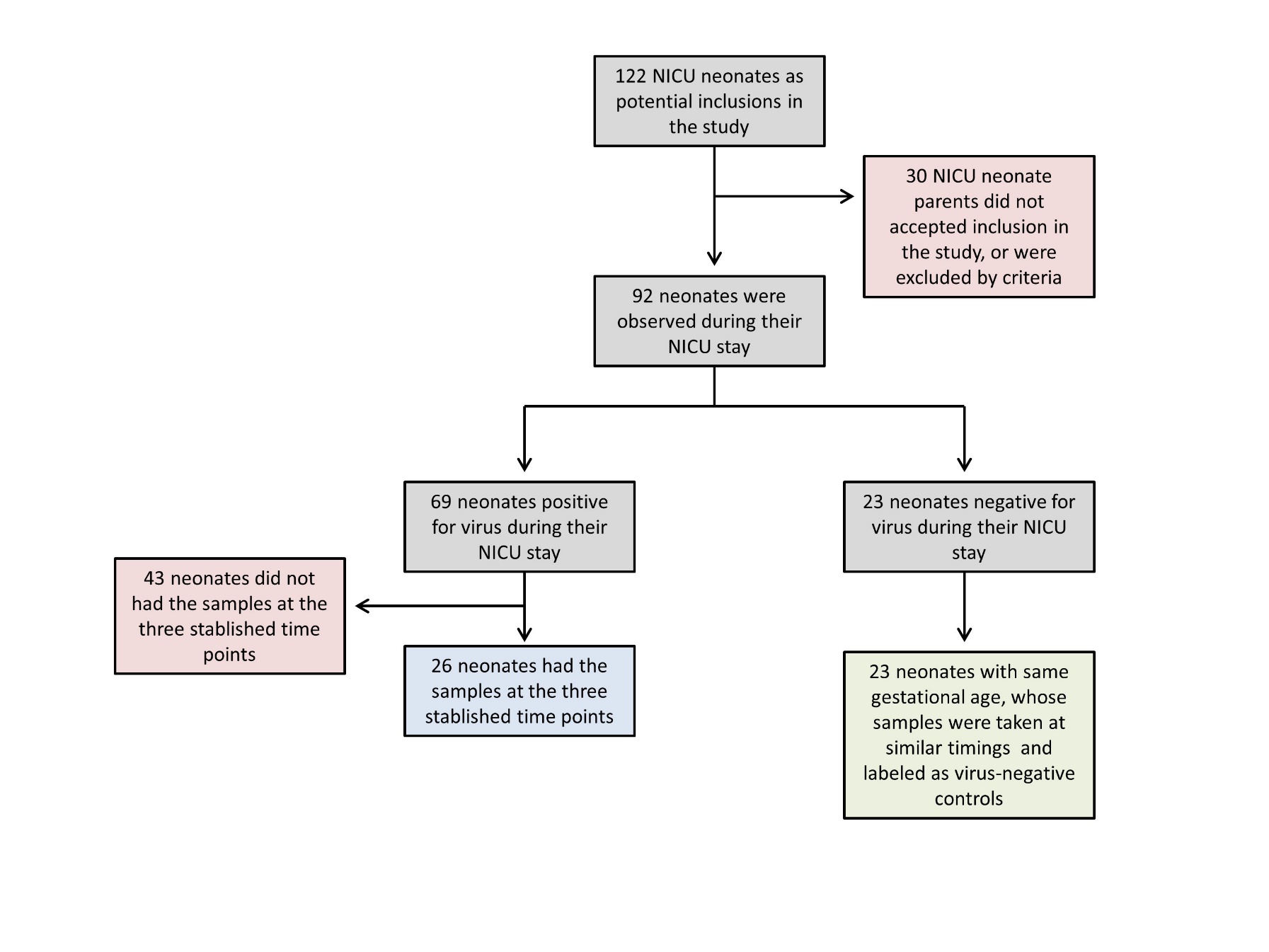
**Supplementary figure S5. Flow chart of neonate numbers in the study.**

**Supplementary figure legends.**

**Supplementary figure 1**. There are no differences at the cytokine and molecular levels due to sex differences. The figure shows protein quantities of filaggrin, VEGF, MIP-1α, MIP-1β, RANTES, HIF-1α, IL-17A, IL-1β, and MCP-1 in samples from virus-negative controls or negative pre-infection, viral positive, and negative post-infection samples from virus-positive premature neonates, further classified depending on sex (males and females) measured by ELISA or Luminex technology. Inter-group analysis was performed using Mann-Whitney Wilcoxon test. Bar graphs represent median and interquartile ranges. *P<0.05 and **P<0.01.

**Supplementary figure 2.** Virus entity does not cause a differential profile of cytokine levels. The figure shows protein quantities of filaggrin, VEGF, MIP-1α, MIP-1β, RANTES, HIF-1α, IL-17A, IL-1β, and MCP-1 in samples from virus-positive premature neonates, further classified depending on viral entity (rhinovirus, n=6; bocavirus [BoV} and/or adenovirus [AdV], n=5; coronavirus, n=5; or other, n=3) measured by ELISA or Luminex technology. Inter-group analysis was performed using using Kruskal-Wallis test with multiple comparisons followed by Dunn’s uncorrected post-test. Bar graphs represent median and interquartile ranges. *P<0.05.

**Supplementary figure 3.** ROC curve representation of the AUC values of individual molecules measured in the NPAs from neonates (a) and for the logistic regression models created by biomarker combination (b) for the differentiation of neonates with viral infection predisposition and those with active infection *versus* the negative non-infected controls. AUC: Area under the curve.

**Supplementary figure 4.** Original represented western blots for study of filaggrin in the NPA cells, showing the samples represented and both protein bands of β-actin (45kDa) and filaggrin (25kDa).

**Supplementary figure 5.** Flow chart which describes the number of neonates that have been considered, excluded, included and selected for the study.
